# Age-associated chemokine receptor expression profiles in human peripheral blood monocyte subsets predict cardiovascular disease risk

**DOI:** 10.3389/fimmu.2026.1749366

**Published:** 2026-02-23

**Authors:** Ravi K. Komaravolu, Nandini Chatterjee, Sunil Kumar, Jessica L. Allen, Christopher Durant, Runpei Wu, Gabriel Valentin-Guillama, Chantel McSkimming, Fabrizio Drago, Angela M. Taylor, Yury I. Miller, Klaus Ley, Coleen A. McNamara, Ahmad Alimadadi, Catherine C. Hedrick

**Affiliations:** 1 Immunology Center of Georgia, Augusta University, Augusta, GA, United States; 2 La Jolla Institute for Immunology, La Jolla, CA, United States; 3 Beirne B. Carter Center for Immunology Research, University of Virginia, Charlottesville, VA, United States; 4 Cardiovascular Division/Department of Medicine, University of Virginia, Charlottesville, VA, United States; 5 Division of Endocrinology, University of California San Diego, San Diego, La Jolla, CA, United States

**Keywords:** Ab-Seq, biomarkers, cardiovascular disease, chemokine receptors, Gensini score, immune aging, monocytes, inflammation

## Abstract

**Background:**

Aging is a major contributor to chronic inflammation and coronary artery disease (CAD), yet how age influences monocyte chemokine receptor expression in relation to disease severity remains incompletely defined.

**Methods and results:**

We performed high-dimensional single-cell antibody sequencing (Ab-Seq) of peripheral blood mononuclear cells from 61 participants (ages 42–78 years) enrolled in the Coronary Assessment of Virginia (CAVA) cohort. Aging was associated with remodeling of monocyte populations, including a reduction in anti-inflammatory classical monocytes and an expansion of immature monocytes. Among younger individuals with severe CAD, intermediate monocyte subcluster iMo_HLA-DR^int^CCR2^low^ was increased, whereas anti-inflammatory classical monocyte cMo_CD33^hi^CD163^hi^CXCR4^+^ was reduced. In older individuals with progressive CAD, further reductions in CCR6^+^ and CXCR3^+^ classical monocytes were observed. Additional flow cytometry validation confirmed decreased CCR6-expressing classical monocytes in older individuals with high CAD burden. Independent of age, CXCR3-expressing intermediate monocytes were significantly increased in individuals with severe CAD. Transcriptomic analysis of CXCR3^+^ intermediate monocytes demonstrated increased expression of C1Q genes compared with CXCR3^low^ cells. Interestingly, chemokine receptor expression also correlated with lipid parameters in older individuals where CCR6 expression on intermediate monocytes positively associated with HDL cholesterol and increased with CAD severity, whereas CXCR3 expression on classical monocytes declined with advancing CAD.

**Conclusions:**

Aging is associated with distinct changes in monocyte chemokine receptor expression that relate to CAD severity. These findings identify age- and disease-associated monocyte immune features that may contribute to CAD progression.

## Introduction

Aging is a major risk factor for a broad spectrum of chronic inflammatory disorders and is closely associated with immune dysregulation in individuals over 65 years of age, a phenomenon often referred to as “inflammaging” (1, 2). Older adults frequently exhibit vascular inflammation and cardiac pathologies, underscoring the intimate link between aging and immune cell function (3–5). Although the prevalence and mortality of coronary artery disease (CAD) rise sharply with age, the distribution and phenotypic characteristics of circulating monocytes across distinct stages of CAD remain incompletely characterized. Elucidating how monocyte phenotype and function evolve with age is therefore essential to uncover mechanisms connecting inflammaging to cardiovascular disease.

Human monocytes are classified into three major subsets based on their surface marker expressions of CD14 and CD16, for example, classical (cMo_CD14^+^CD16^-^), intermediate (iMo_CD14^+^CD16^+^), and non-classical (nMo_CD14^-^CD16^+^), each exhibiting distinct transcriptional programs (6). Previous studies in healthy individuals have demonstrated that both the composition and functional state of these monocyte subsets undergo significant changes with increased age (7). A recent study by Cao et al. using flow cytometry demonstrated that phenotypic and functional alterations in monocyte subsets are key contributors to age-related inflammation (8). We and others have previously shown that alterations in some circulating monocytes and their activation states are strongly linked to CAD progression (5, 8–11), and multiple studies further implied that a shift toward more pro-inflammatory monocyte subsets correlates with impaired vascular function and accelerated plaque development (7, 12, 13). Beyond identifying monocyte abundance, it is equally important to understand the polarization of chemokine receptor signaling in monocytes, as these receptors directly influence vascular inflammation and atherosclerotic plaque development (14, 15). Thus, identifying human monocyte-specific C-C and C-X-C chemokine receptor expression profiles in CAD is crucial since these receptors and their ligands are critical regulators of monocyte migration and interactions with lymphocytes and the vascular endothelium (7, 16). Of note, altered expression of some of these receptors has been implicated in mouse atherosclerosis models (11), yet a detailed analysis of aged human monocytes in vascular diseases at single-cell resolution has been lacking. Although the roles of some of the C-C and C-X-C chemokine family members are well established in non-myeloid cells, particularly in the context of cardiovascular inflammation in both human and animal models (17–19), their roles in monocyte subtypes remain less defined. Previous studies observed that the recruitment of CXCR3^+^, CCR2^+^, and CCR5^+^ monocytes to activated endothelium is a hallmark of atherosclerosis, contributing critically to inflammatory amplification, and plaque progression (18–20). Recently, we identified that intermediate monocytes (iMo) capture clinically relevant heterogeneity in cardiovascular disease. We found that the frequency of the iMo_HLA-DR^+^CXCR3^+^CD206^+^ subset positively correlated with CAD severity, with a stronger association in females (9). Although our previous findings identify CXCR3 as a key iMo-specific marker in CAD progression, its distribution in across aging remains unclear. Studies using Antibody-based Single-Cell RNA Sequencing (Ab-Seq) methods from our group and others have demonstrated the diversity of monocytes present in human peripheral blood mononuclear cells (PBMCs) in our Coronary Assessment in Virginia (CAVA) cohort (5, 9, 21). While previous studies identified some pathways governing age-associated immune cell dysfunction (22, 23), the exact frequencies of specific monocyte subsets and their involvement in such inflammatory signaling pathways across age remain poorly understood.

The aim of this study was to examine whether chemokine receptor expression profiles on monocytes vary with age and CAD risk. To address this, we employed high-dimensional Ab-Seq profiling to comprehensively examine age- and coronary artery disease-associated changes in chemokine receptor expression on circulating monocytes. Specifically, we examined five C–C (CCR2, CCR4, CCR5, CCR6, and CCR7) and three C–X–C (CXCR3, CXCR4, and CXCR5) receptors on monocyte subsets across age (younger and older) and CAD (low and high) and further correlated receptor expression patterns with key clinical parameters. Overall, our findings reveal age-related shifts in chemokine receptor expression that define distinct monocyte phenotypes associated with inflammaging.

## Methods

### Human subjects

The study enrolled subjects with low to high coronary artery disease based on their Gensini score (calculated by quantitative coronary angiography), aged between 42 and 78 years, from the Coronary Assessment in Virginia (CAVA) cohort. In this study, older age was defined as ≥65 years, consistent with commonly used thresholds in clinical and epidemiological research (24) https://www.nih.gov/nih-style-guide/age. Participants were recruited through the Cardiac Catheterization Laboratory at the University of Virginia Health System in Charlottesville, VA, USA, following physician referrals for medically necessary cardiac angiography. Written informed consent was obtained from all participants before enrollment, and the study received approval from the UVA Human Institutional Review Board (IRB No. 15328). Peripheral blood mononuclear cells were collected from all participants as described in our earlier studies (9, 10, 21), and clinical features, including CAD severity (Gensini score), sex, age, statin treatment, and diabetes status, were documented (**Supplementary Tables 1A–C**). PBMC Samples that failed to meet quality control criteria, including cell viability, were excluded from the analysis (n=4), and the final study analysis included a total of 61 samples.

### Quantitative Coronary Angiography

The methods for performing Quantitative Coronary Angiography (QCA) and the calculation of the Gensini score have been outlined in detail in studies conducted by our group (9, 10, 21). Subjects with a Gensini score ≥32 were classified as high CAD, and subjects with a Gensini score ≤6 were classified as low CAD. Comprehensive analyses of Gensini scores highlighting statistically significant differences by CAD severity (Low CAD, High CAD) and age group (Younger and Older), together with associated demographic and clinical characteristics, are presented in **Supplementary Tables 1A** through **1C**.

### PBMC sample preparation for Ab-Seq, library preparation, and sampling

Peripheral blood was collected into BD K2 EDTA tubes and processed at room temperature within 60 min, as described previously (5, 9). PBMCs were isolated by Ficoll-Paque PLUS density gradient centrifugation using SepMate-50mL tubes (#85460, StemCell Technologies) following the manufacturer’s instructions. Cells were counted and cryopreserved in 90% FBS + 10% DMSO. To minimize batch effects, we processed at least eight samples in parallel, thawed them at 37°C, centrifuged them at 400×g for 5 min, and resuspended them in cold staining buffer. Viability and counts were measured using the BD Rhapsody Scanner. The scRNA sequencing was performed using Antibody-seq (Ab-seq) on the BD Rhapsody high-throughput single-cell analysis platform, in which antibody-derived tags (ADT) and single-cell gene expression libraries are simultaneously captured from the same individual cells. We used a customized 51 antibody, 487 immune gene panel, including molecular barcoding, an approach we have previously published and validated in multiple studies (9, 10, 21, 25).

Of 65 samples, 61 met QC criteria (viability > 80%). Each sample was barcoded with the BD Sample Multiplexing Kit, washed, pooled, stained with a 49 validated antibody surface marker panel and loaded onto Rhapsody nanowell plates using four samples per plate. Library preparation was performed as previously described (9, 10). Briefly, primed plates were loaded with cells at 800–1,000 cells/μL and incubated on a thermomixer (37°C, 1,200 rpm, 20 min) for reverse transcription. Exonuclease I treatment was followed (37°C, 30 min), after which the plate was heated to 80°C for 20 min. cDNA libraries were prepared according to the BD protocol described by Vallejo et al. (16). Library quality and quantity were assessed using the TapeStation and Qubit systems. Sequencing generated ~60,600 reads per cell on the Illumina NovaSeq platform, with pooling and depth optimized for S1 and S2 100-cycle kits. FASTA/FASTQ files were processed through the Seven Bridges Genomics pipeline. RNA and ADT assays were normalized using the LogNormalize (scale factor 10,000) and centered log-ratio (CLR) methods, respectively. Doublets were filtered with the DoubletFinder package (26).

### Multimodal data integration and clustering

Analyses were performed in R v4.1.0 using Seurat v4 (27). All 49 ADT features and the top 200 variable RNA features identified using the “vst” method were scaled and subjected to PCA. The first 20 of 50 components were used for batch correction with Harmony v0.1.1 (28). Batch-corrected embeddings were integrated using FindMultiModalNeighbors (WNN), followed by Louvain clustering at a resolution of 1, and t-distributed stochastic neighbor embedding (t-SNE) made with ADT expression was used to visualize the clusters. Clusters with fewer than 100 cells were excluded. For myeloid cells, non-expressed markers were removed, and both modalities were normalized and analyzed by PCA, Harmony, FindMultiModalNeighbors, and FindClusters (resolution=1.7). The clusters were annotated into three major and 13 smaller subsets of monocytes.

### Correlation analyses of monocytes

Spearman’s rank correlation was performed using the “cor.test” function from the stats package in R (29). This analysis was used to assess associations between age, clinical variables, and chemokine receptor expression across monocyte subsets, as well as relationships between monocyte cluster proportions and either age or Gensini score. For each patient average expression levels of chemokine receptors (CXCR3, CXCR4, CXCR5, CCR2, CCR4, CCR5, CCR6, and CCR7) were calculated separately within the specified monocyte subsets. Correlation analysis results and expression patterns were visualized using either radar plots, heatmaps and/or bubble plots.

### Flow cytometry analysis of chemokine receptors in a validation cohort

Matched PBMC samples used in the previous Ab-seq study were obtained for validation experiments (n=7 high CAD and n=5 low CAD). The detailed staining protocol was described previously (9). Briefly, Samples were collected from older individuals with high and low CAD. Thawed PBMCs were washed with 10 mL of 1× PBS and centrifuged at 400 g for 10 minutes at room temperature. Cell viability and counts were measured using a hemocytometer and a Cellometer (Nexcelom). At least 1.5 × 10^6^ cells per well were incubated with Human TruStain FcX™ (BioLegend) for 10 minutes at 4°C, followed by Live/Dead Blue staining (Invitrogen) for 30 minutes at 4°C. Cells were then washed and stained with a surface antibody panel for 40 minutes at 4°C. Anti-CD3, Anti-CD19, and Anti-CD56 antibodies were used to exclude T, B, and NK cells. Monocyte subsets were identified as CD14^+^CD16^−^ (cMo), CD14^+^CD16^+^ (iMo), and CD14^−^CD16^+^ (nMo). Data were collected on a Cytek Aurora spectral flow cytometer and analyzed with FlowJo (BD Biosciences). Fluorescence-minus-one (FMO) controls from healthy donors were used to set gating.

### Statistical analysis

Differentially expressed genes (DEGs) were identified using the FindMarkers function in Seurat (test.use = “MAST”, logfc.threshold = 0.25 or 0.1, min.pct = 0.25 or 0.1). The logfc.threshold and min.pct values used for each analysis are mentioned in the legends. Genes with adjusted p< 0.05 were considered significant. Pathway analysis was performed with the clusterProfiler package (30) using Gene Ontology Biological Process terms, and separately for up- and down-regulated DEGs.

A generalized linear mixed model (GLMM) was used to evaluate differences in myeloid cluster proportions, while accounting for both fixed and random sources of variability, using the lme4 package v1.1-31 (31). This modeling framework allows group-level comparisons while accounting for variability across individuals and adjusting for patient-level factors that may differ between groups. Patient, sex, diabetes, and statin use were considered as random effects when comparing Older_LowCAD versus Younger_LowCAD, Younger_highCAD versus Younger_LowCAD and Older_HighCAD versus Older_LowCAD. We analyzed the cMo_CCR6 validation data using the Mann-Whitney U test in Prism (GraphPad Software, Inc. version 10.1.0), data shown as mean ± SE (*p-value* < 0.05 was considered statistically significant).

To visualize the relationship between marker expression and Gensini score, we used smooth plots. To assess whether this association differed by age, we fitted generalized additive models (GAMs) using ‘mgcv’ R package (32) with either age-specific or shared smooth terms for Gensini score, while accounting for baseline differences between age groups. Models were compared using ANOVA (F-tests), and the resulting p-values were used to assess age-dependent effects.

## Results

### C-C and C-X-C chemokine surface marker expression profiles in human monocytes

**Figure 1:**
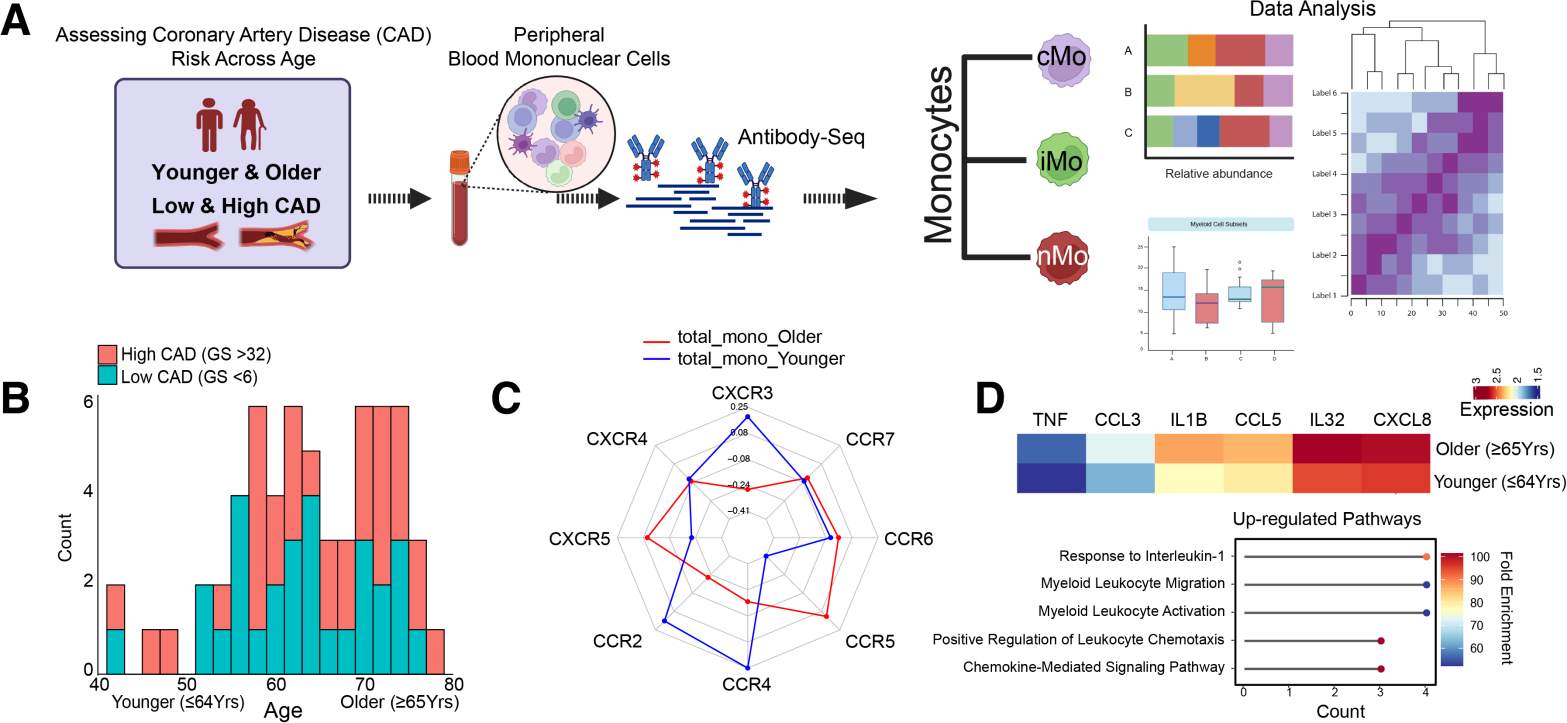
Study design. C-C and C-X-C chemokine surface marker expression profiles in human monocytes. **(A)** Schematic illustrating the study design. The Coronary Assessment of the Virginia Cohort (CAVA) was used to analyze monocyte subsets from PBMCs of younger and older individuals with low and high coronary artery disease (CAD) risk, followed by Antibody-Seq profiling. Figure generated with BioRender (https://biorender.com, accessed on September 2025) **(B)** Age distribution of participants stratified by cardiovascular disease risk based on their Gensini scores (GS); colors indicate high (GS≥32) and low (≤6) CAD groups. **(C)** Radar plot showing correlation values between age and average chemokine receptor expression across younger and older individuals. CCR5 expression in the younger group showed a significant negative correlation with age (*r= −0.41, p-value= 0.032*) **(D)** Heatmap of differentially expressed inflammatory genes (log2FC = 0.1 and min.pct = 0.1) and corresponding GO term enrichment demonstrating increased cytokine and chemokine-mediated signaling and leukocyte migration pathways in older subjects (Age ≥65yrs).

We collected peripheral blood mononuclear cells (PBMC) samples from 61 subjects aged between 42 and 78 years who were recruited in the Coronary Assessment in Virginia (CAVA) cohort (9). We performed single-cell high-dimensional antibody sequencing (Ab-Seq) on PBMCs from each subject using the BD Rhapsody platform to comprehensively study changes in monocyte-specific chemokine receptor markers across age and coronary artery disease (CAD). (**Figure 1A**). Subjects were stratified by age, older (≥65 years, n=33) and younger (≤64 years, n=28), and by CAD severity using the Gensini score, where a GS ≤6 =low CAD and a GS ≥32 =high CAD (**Figure 1B**). Covariates included sex, diabetes status, smoking, and statin use. Comprehensive clinical data pertaining to low CAD risk individuals (older, mean age 70.3 ± 4.0; and younger, mean age 56.7 ± 5.3, *p-value* < 0.0001) and older individuals with CAD group (high CAD, mean GS 62.8 ± 35.8 and low CAD, mean GS 3.3 ± 2.4, *p-value* < 0.0001). Detailed clinical characteristics for all age and CAD groups were shown in **Supplementary Tables 1A to 1C**. Our Antibody-Seq custom panel (9) included antibodies to detect five C-C chemokine receptors (CCR2, CCR4, CCR5, CCR6, and CCR7), and three C-X-C chemokine receptors (CXCR3, CXCR4, and CXCR5).

To determine whether chemokine receptor expression showed age-related patterns in monocytes, we first analyzed correlations between chemokine receptor expression levels and age across all circulating monocytes, independently of CAD status. Spearman correlation analysis of the eight chemokine receptors, visualized in a radar plot, revealed distinct age-associated trends (**Figure 1C**). Except for CCR5 in younger individuals (*r=-0.41, p-value=0.03*), all other correlations did not reach statistical significance. CCR5 and CXCR5 expression tended to increase with age, whereas expression of CXCR3, CCR2, and CCR4 was more highly correlated with younger individuals among the total monocyte population. The transcriptional profile of monocytes in older individuals (≥65 years) showed upregulation of inflammatory mediators, including *IL1B, CCL5, IL32*, *TNF, CCL3*, and *CXCL8*. Concomitantly, pathway enrichment analysis showed upregulation of pathways related to interleukin-1 signaling, myeloid leukocyte migration, and chemokine-mediated signaling in monocytes from older subjects (**Figure 1D**; **Supplementary Table 1D**). Collectively, these findings indicate that older age is associated with an increase in systemic pro-inflammatory genes in circulating monocytes.

### Chemokine receptor expression patterns in three major monocyte populations

**Figure 2:**
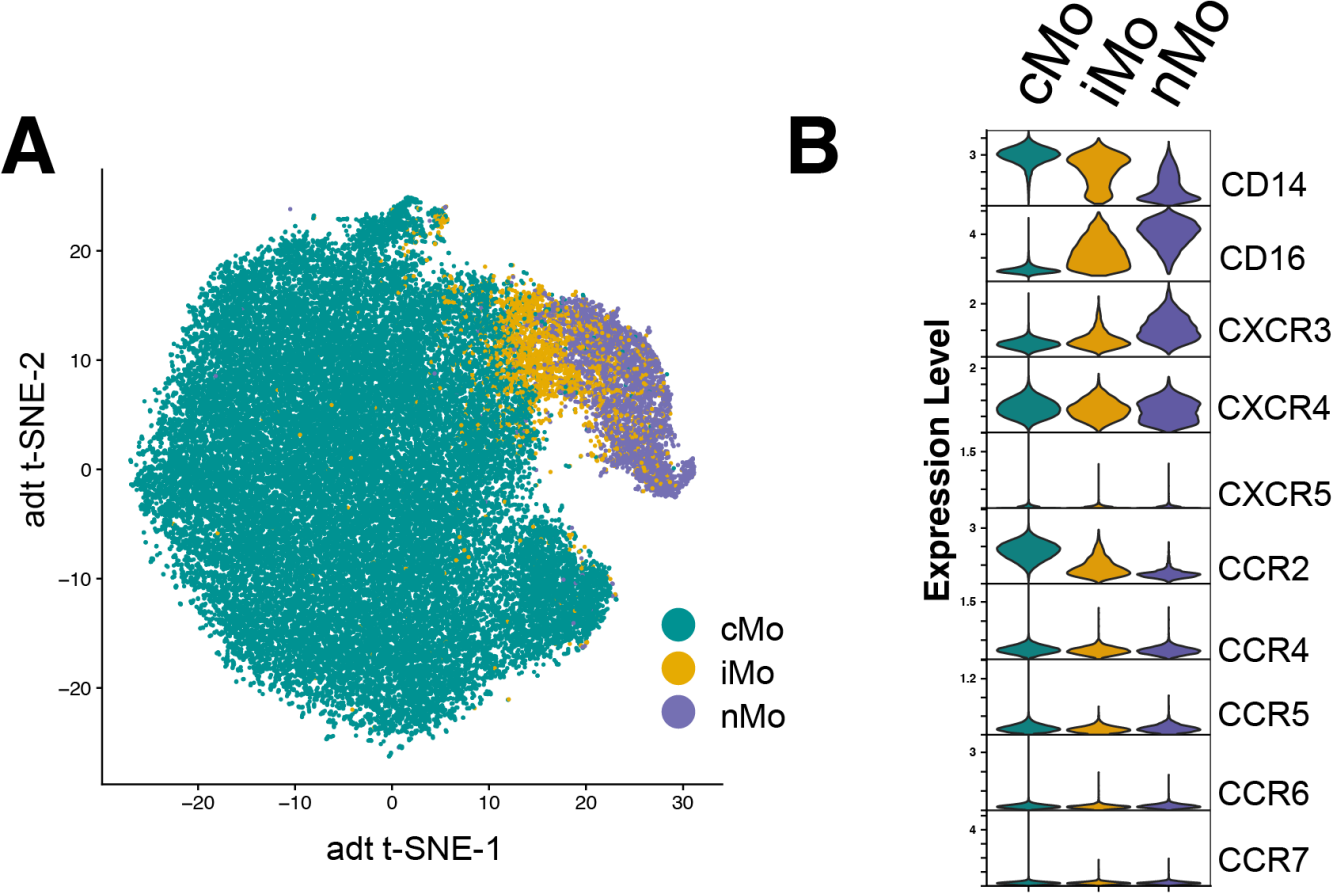
Chemokine receptor expression patterns in three major monocyte populations.**(A)** t-SNE plot showing clustering of classical (cMo), intermediate (iMo), and nonclassical (nMo) monocytes based on Antibody-Seq data. **(B)** Violin plots illustrating scaled protein expression of key chemokine receptors (CXCRs and CCRs) and canonical monocyte markers (CD14, CD16) across the three major monocyte subsets, cMo, iMo, and nMo.

Next, we asked whether individual chemokine receptors display age-dependent expression patterns within the three major monocyte populations (classical, intermediate, and non-classical). t-SNE analysis of 45,173 monocytes from all subjects revealed three distinct clusters corresponding to classical (cMo_CD14^+^, 38,953 cells), intermediate (iMo_CD14^+^CD16^+^, 2,599 cells), and non-classical (nMo_CD16^+^, 3,621 cells) subsets, showing clear separation among populations (**Figure 2A**; **Supplementary Figure S1**). In the t-SNE projection, each color denotes a distinct monocyte subset derived from classical (cMo), intermediate (iMo), or nonclassical (nMo) populations, as defined by surface expression of the canonical markers CD14 and CD16 (**Figures 2A, B**). Their distinct chemokine receptor protein (**Figure 2B**) and their corresponding gene expression profiles are shown in **Supplementary Figure S4A**. We observed CCR2 expression as the highest in cMo which declined gradually in iMo and nMo, whereas CXCR3 expression increased along the transition from cMo to nMo, indicating a shift in chemokine receptor distribution in cMo and nMo (**Figure 2B**). Together, these findings suggest coordinated, C-C and C-X-C receptor-specific expression across the three major monocyte subsets.

### Chemokine receptor expression patterns across age in monocyte subclusters in low CAD risk individuals

**Figure 3:**
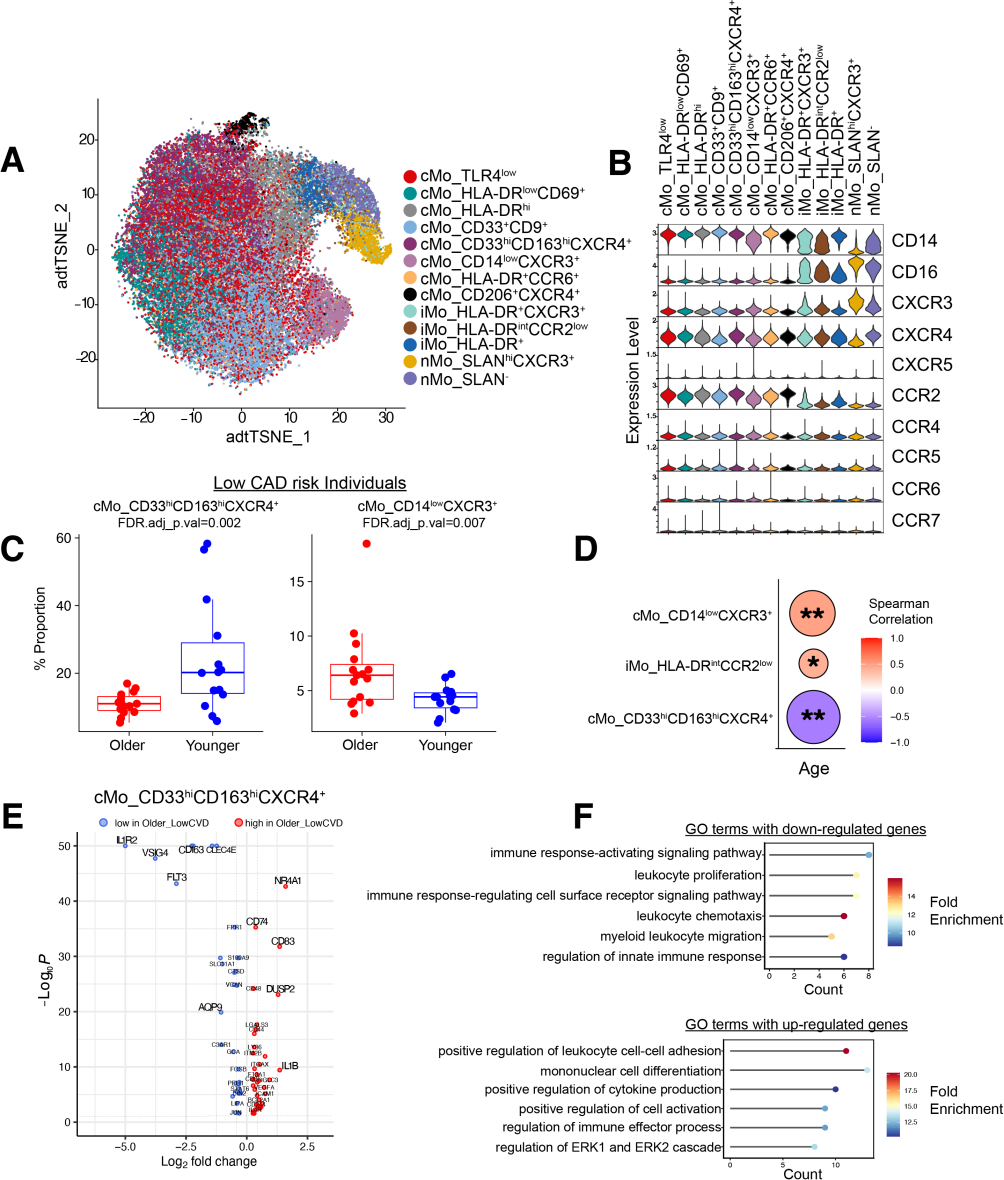
Chemokine receptor expression patterns across age in monocyte subclusters in low CAD risk individuals. **(A)** t-SNE plot showing distinct monocyte subclusters defined by surface marker and chemokine receptor expression profiles, with each subcluster represented by a unique color. **(B)** Violin plots showing scaled protein expression of key monocyte and chemokine receptor markers across the identified subclusters; classical monocyte marker CD14 and nonclassical monocyte marker CD16 are also included. **(C)** Box plots showing significant age-associated differences in the proportions of cMo_CD33^+^CD163^+^CXCR4^+^ (*FDR adj. p-values = 0.002*) and cMo_CD14^low^CXCR3^+^ (*FDR adj. p-values = 0.007*) subclusters in low CAD risk individuals. The p-values were calculated using a GLMM model (see methods). **(D)** Spearman correlation analysis depicting the relationship between age and proportions of significantly altered monocyte subclusters. *: p-value < 0.05, **: p-value < 0.01. **(E)** Volcano plot displaying differentially expressed genes (log2FC = 0.25 and min.pct = 0.25) in cMo_CD33^+^CD163^+^CXCR4^+^ cells from older versus younger individuals. **(F)** Gene Ontology (GO) enrichment analysis of up- and down-regulated genes, highlighting pathways related to leukocyte activation, migration, and immune signaling in the subset shown in 3E.

To investigate whether chemokine receptor expression profiles contribute to monocyte heterogeneity, we subclustered the monocytes from **Figure 2A**. At single-cell resolution, distinct expression patterns of CD14 and CD16 allowed us to classify each monocyte subcluster precisely. Our analysis identified 13 subclusters in total, including eight cMo, three iMo, and two nMo populations (**Figure 3A**). Consistent with earlier observations (**Figure 2B**), CCR2 expression was strongly enriched across all cMo clusters, whereas CXCR3 expression was moderately expressed in iMo and peaked in nMo, in the nMo_SLAN^hi^CXCR3^+^ subset (**Figures 3B**, **2B**). Monocyte subcluster-specific gene expression profiles (scaled average expression) were shown in **Supplementary Figure S4B**. To further examine how aging affects these monocyte subclusters in the absence of CAD, we focused on individuals who were classified as low CAD risk (Gensini score ≤6). We then compared the proportions of all monocyte subclusters between younger and older groups within this low-risk cohort (**Supplementary Figures S2A, S2B**). As shown in **Figure 3C**, older subjects exhibited a significant decline in cMo_CD33^hi^CD163^hi^CXCR4^+^ cells (FDR-adjusted *p-value* = 0.002) and a corresponding expansion of cMo_CD14^low^CXCR3^+^ immature classical monocytes subsets (*FDR-adjusted p-value =0.007*). Additionally, Spearman correlation analysis confirmed these age-associated trends, where cMo_CD14^low^CXCR3^+^ (*r=0.48, p-value=0.009*) and iMo_HLA-DR^int^CCR2^low^ *(r* = 0.41, *p-value* = 0.028) subsets were positively correlated with age, whereas cMo_CD33^hi^CD163^hi^CXCR4^+^ cells showed a strong negative correlation (*r*=–0.56, *p-value* = 0.001) (**Figure 3D**). The frequencies of the remaining monocyte subclusters and their age-associated correlations were not statistically significant (**Supplementary Figures S2B**, **S3C**). Transcriptomic profiling of these age-associated subclusters revealed striking differences. Among all populations identified, only the cMo_CD33^hi^CD163^hi^CXCR4^+^ subset displayed significant transcriptomic differences between ages, with 68 differentially expressed genes found in older individuals (**Figure 3E**; **Supplementary Table 1E**). Additional clusters with fewer differentially expressed genes are shown in **Supplementary Figure S2D**. Functional pathway enrichment analysis of the subcluster indicated downregulation of immune response pathways related to leukocyte proliferation and migration, and upregulation of processes associated with mononuclear differentiation, leukocyte adhesion and regulation of ERK1 and ERK2 cascade in older low CAD individuals (**Figure 3F**). Collectively, these findings demonstrate age-dependent reprogramming of monocyte subclusters, characterized by a coordinated decrease in anti-inflammatory cMo_CD33^hi^CD163^hi^CXCR4^+^ and an expansion of CD14^low^ immature cMo_CD14^low^CXCR3^+^ monocytes, highlighting probable functional remodeling of circulating monocytes with increasing age.

### Monocyte subset–specific chemokine receptor expression patterns across CAD and age

**Figure 4:**
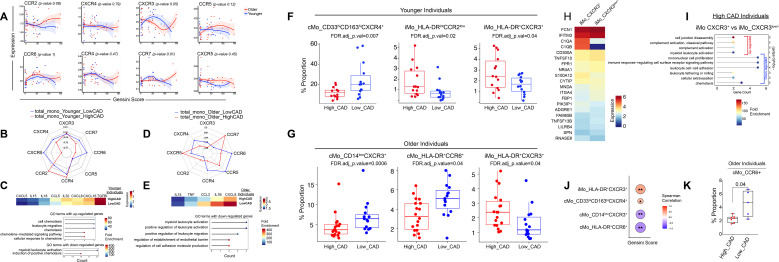
Monocyte subset–specific chemokine receptor expression patterns across CAD and age.**(A)** Smooth plots showing chemokine receptor expression across Gensini scores (x-axis), with data points colored in red for older and blue for younger individuals. P-values for age-dependent differences were calculated via F-tests comparing Generalized additive model (GAM) models with and without age-specific smooths. Radar plot showing correlation values between patient age and patient-level average chemokine receptor expression in total monocytes from younger individuals **(B)** and older individuals **(D)** with low versus high CAD risk. The trends shown here are descriptive, as no statistically significant correlations were detected. Heatmaps showing differential expression of inflammatory cytokine genes in younger **(C)** and older **(E)** high versus low CAD younger groups, with corresponding GO terms highlighting downregulated pathways related to leukocyte activation and migration. (**F**) Box plots depicting significant differences in the proportions of cMo_CD33^hi^CD163^hi^CXCR4^+^ (*FDR.adj_p.value=0.007*), iMo_HLA-DR^int^CCR2^low^ (*FDR.adj_p.value=0.02*), and iMo_HLA-DR^+^CXCR3^+^ (*FDR.adj_p.value=0.04*) subclusters between high and low CAD risk groups in younger individuals. **G**) Box plots depicting significant differences in the proportions of cMo_CD14^low^CXCR3^+^ (*FDR.adj_p.value=0.0006*), cMo_HLA-DR^+^CCR6^+^ (*FDR.adj_p.value=0.04*), and iMo_HLA-DR^+^CXCR3^+^ (*FDR.adj_p.value=0.04*) subclusters between high and low CAD risk groups in older individuals. The *p-values* were calculated using a GLMM model, which is explained in the methods section. **H**) Transcriptomic profiles of iMo_CXCR3^+^ and iMo_CXCR3^−^ cells from individuals with high CAD risk across all age groups. Data analysis parameters were set to Min.pct_0.1_logFC.threshold 0.1 in the Seurat package. To define the iMo_CXCR3^+^ cells transcriptional signature, both Protein and RNA expression profiles of CXCR3 were first identified to separate iMo_CXCR3^+^ and iMo_CXC3^low/-^ populations. For iMo_CXCR3^low/-^ cells, iMo_HLA-DR^^+^^ and iMo_HLA-DR^int^CCR2^low^ cells were combined and compared with iMo_CXCR3^+^ cells to identify differentially expressed genes and associated pathways. **I)** Gene Ontology (GO) terms associated with up- and down-regulated genes identified in the heatmap shown in panel 4H. Pathway significance is represented as −log_10_ (*adjusted p values*). Fold enrichment is indicated by the color scale in the lollipop plots. **(J)** Spearman correlation analysis showing associations between Gensini score and their significant monocyte subclusters. **(K)** Validated experimental data using flow cytometry showing a reduced proportion of CD14^+^ cMo_CCR6^+^ cells in high CAD (n=7) compared to low CAD (n=5) older individuals (*p-value* = *0.04*). *: p-value < 0.05, **: p-value < 0.01, and ***: p-value < 0.001.

Having established that aging alters some of the observed C–C and C–X–C chemokine receptor expression profiles on monocyte subsets, we next asked whether these changes also reflect disease progression across age. To address this, we first observed chemokine receptor expression across all monocytes and stratified participants by Gensini score. Nonlinear trend analyses using LOESS fitting revealed CAD severity–dependent variations in multiple chemokine receptor expression across total monocytes in both younger and older individuals. This analysis specifically showed an upward trend in CXCR3 (*p-value=0.05*) and CCR7 (*p-value=0.01*) expression associated with increased Gensini score in older compared with younger individuals with high CAD (**Figure 4A**). To better understand CAD-associated inflammatory shifts in chemokine receptor expression. We examined the relationship between age and patient-level average chemokine receptor expression in monocytes among younger (**Figure 4B**) and older adults (**Figure 4D**) stratified by low and high CAD risk. Although no associations reached statistical significance, younger individuals with high CAD exhibited positively trended correlations, specifically between CCR2 and CCR4 expression and high CAD burden (**Figure 4B**), suggesting early inflammatory remodeling of monocyte chemokine responsiveness. Consistent with these observations, differential gene expression and pathway analyses in younger individuals with high CAD revealed a statistically significant upregulation of *TGFB1, CXCL16, IL32, CCL5*, and *IL15*. These transcriptional changes were accompanied by enrichment of pathways related to chemokine-mediated signaling and cellular responses to chemokines, indicating heightened inflammatory activation in this group (**Figure 4C**; **Supplementary Table 1F**). In contrast, older individuals with high CAD burden exhibited downregulation of key inflammatory genes, including *CXCL8, IL1B*, and *CCL3* (**Figure 4E**). Pathway enrichment analysis in this group highlighted activities associated with regulation of the endothelial barrier and negative regulation of cell adhesion molecule production, suggesting attenuation of pro-inflammatory and leukocyte–endothelial interaction pathways with age (**Figure 4E**; **Supplementary Table 1G**). Together, these findings suggest age-dependent divergence in monocyte inflammatory programming in CAD, with enhanced chemokine expression-driven inflammatory signaling in younger individuals and relative dampening of vascular wall and pro-inflammatory effector pathways in older adults.

We next assessed the frequencies of individual monocyte subclusters in both younger and older individuals stratified by their CAD status. In younger individuals with high CAD, we observed a significant reduction in cMo_CD33^hi^CD163^hi^CXCR4^+^ cells (*FDR-adjusted p-value = 0.007*), and an increase in iMo_HLA-DR^int^CCR2^low^ populations (*FDR-adjusted p-value* = 0.02) (**Figure 4F**). In older individuals with high CAD, the frequencies of cMo_CD14^low^CXCR3^+^ (*FDR-adjusted p-value* = 0.0006) and cMo_HLA-DR^+^CCR6^+^ (*FDR-adjusted p-value* = 0.04) subsets were significantly reduced (**Figure 4G**). Interestingly, iMo_HLA-DR^+^CXCR3^+^ is the only population which was expanded by approximately two-fold in individuals with high CAD, an effect that was independent of age (*FDR-adjusted p-value* = 0.04), shown in **Figures 4F, G**, *right panels*. The remaining subclusters and their CAD-associated proportions in both younger and older individuals that were not statistically significant are presented in **Supplementary Figures S3A, S3B, S3C**.

To further characterize the transcriptomic profile underlying the age-independent expansion of the iMo_HLA-DR^+^CXCR3^+^ cluster in individuals with high CAD, we compared gene expression patterns between iMo_CXCR3^+^ and iMo_CXCR3^low/^−^^ cells. Differential gene expression analysis revealed significantly increased expression of the complement genes C1QA and C1QB in iMo_CXCR3^+^ cells (**Figure 4H**; **Supplementary Table 1H**). Pathway enrichment analysis indicated that CXCR3^+^ monocytes were transcriptionally enriched for pathways related to classical complement activation and leukocyte-mediated immunity. In contrast, genes downregulated in CXCR3^+^ relative to CXCR3^low/−^ cells were enriched for Gene Ontology terms associated with leukocyte tethering, rolling, and cell–cell adhesion, highlighting a distinct transcriptional signature associated with CXCR3 expression in intermediate monocytes (**Figure 4I**).

Next, we found that iMo_HLA-DR^+^CXCR3^+^ (r=0.46, *p-value* = 0.006) and cMo_CD33^hi^CD163^hi^CXCR4^+^ (r=0.36, *p-value* = 0.04) subsets showed robust positive associations with CAD severity, whereas cMo_CD14^low^CXCR3^+^ (r=–0.44, *p-value* = 0.009) and cMo_HLA-DR^+^CCR6^+^ (r=–0.50, *p-value* = 0.003) subsets were negatively correlated with CAD (**Figure 4J**). We validated using flowcytometry that there was a ~2.5-fold reduction in cMo_CCR6^+^ monocyte frequencies (*p-value* = 0.04) in additional older high CAD group from CAVA (n=7 high CAD, n=5 low CAD) (**Figure 4K**), consistent with our earlier Ab-Seq data (**Figure 4G**, *middle panel cMo_HLA-DR^+^CCR6^+^*). Overall, these results show that chemokine receptor expression profiles are varied in monocyte subsets of older adults with progressive CAD, highlighting a link between monocyte heterogeneity and disease severity.

### Monocyte chemokine receptor profiles and their associations with cardiovascular risk factors in aged individuals

**Figure 5:**
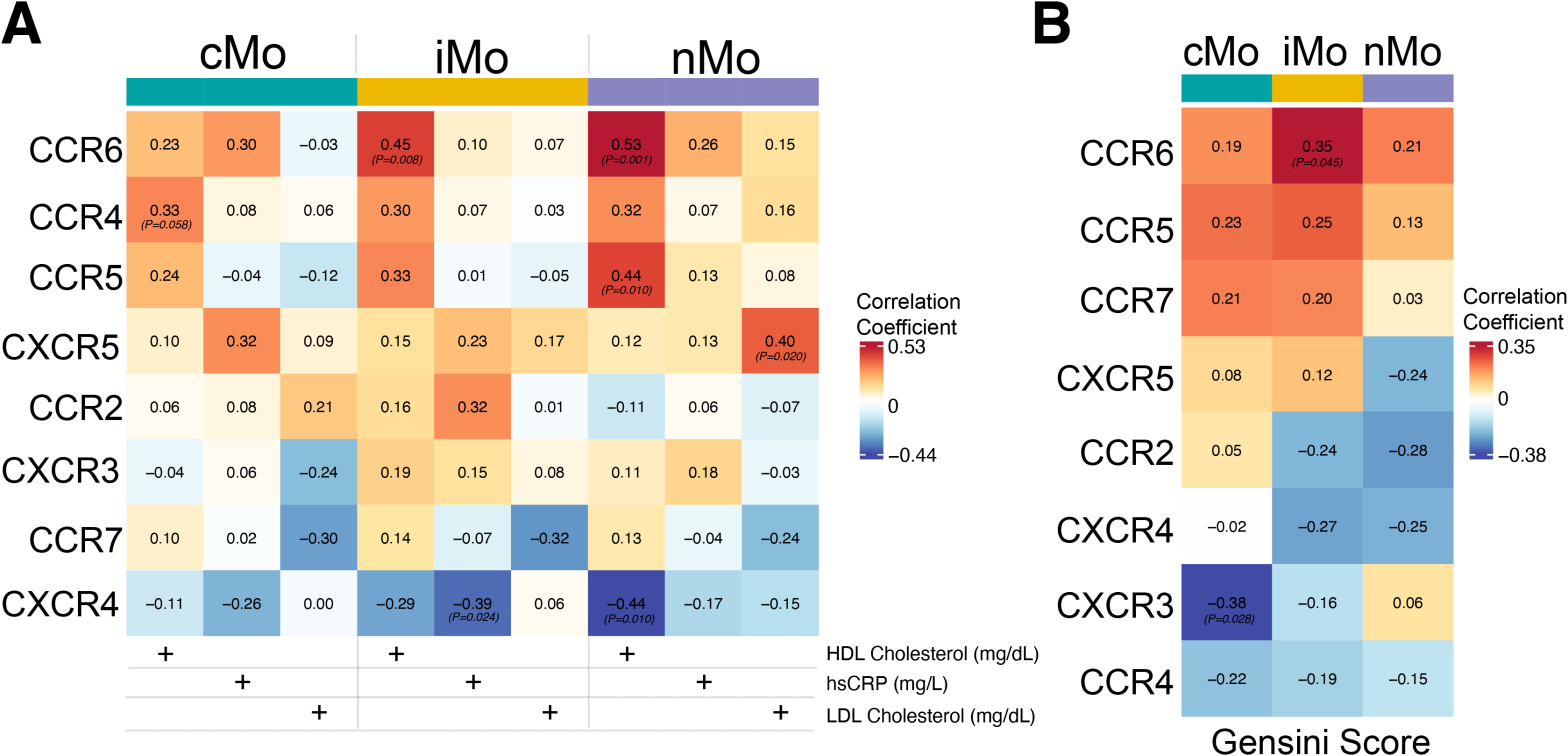
Correlation of chemokine receptor expression with clinical parameters and CAD severity across major monocyte subsets in aged individuals. **(A)** Heatmap showing correlation coefficients between average chemokine receptor expression (C-C and C-X-C family members) in the monocyte subsets and circulating clinical parameters, including HDL cholesterol, hsCRP, and LDL cholesterol, across classical (cMo), intermediate (iMo), and nonclassical (nMo) monocyte subsets. Significant correlations are indicated by p-values. **(B)** Heatmap depicting correlations between average chemokine receptor expression in the monocyte subsets and Gensini scores across three major monocyte subsets, highlighting subset-specific associations between receptor expression and CAD severity.

Finally, to understand how inflammaging-related immune alterations associate with clinical parameters, we examined the relationship between chemokine receptor expression across the three major monocyte subsets and plasma markers of cardiovascular disease progression in older individuals. Using Spearman correlation analyses with systemic risk factors from the same subjects, we found positive associations of chemokine receptors with plasma LDL- and HDL-cholesterol and the inflammatory marker hsCRP (High-Sensitivity C-Reactive Protein) in older subjects within our cohort (**Figure 5A**). Many of the C-C chemokine receptors were positively correlated with plasma HDL-Cholesterol levels, including iMo-CCR6 (*r=0.45*, *p-value =0.008*), nMo-CCR6 (*r=0.53*, *p-value=0.001*), and nMo-CCR5 (*r=0.44*, *p-value =0.010*), and with a modest trend for cMo CCR4 (*r=0.33*, *p-value=0.058*). The C-X-C chemokine receptor CXCR5 is correlated positively with only LDL-Cholesterol in non-classical monocytes (*r=0.40*, *p-value=0.020*). Moreover, CXCR4 expression revealed a significant negative correlation with hsCRP levels for both iMo (r*=0.39*, *p-value=0.024*) and nMo (*r=0.44*, *p-value=0.010*) subsets. Additional analysis of monocyte subsets with CAD severity (Gensini score) showed distinct associations, in which CCR6 expression in intermediate monocytes positively correlated with Gensini Score (*r=0.35*, *p-value=0.045*), whereas CXCR3 expression in classical monocytes showed a strong negative correlation (*r=–0.38*, *p-value=0.028*) (**Figure 5B**). These findings suggest that selective remodeling of chemokine receptor expression within distinct monocyte subsets is likely linked to clinical cardiovascular risk parameters, including plasma cholesterol profiles and inflammatory markers in older individuals.

## Discussion

Aging remodels immune cell functions (8), and our findings here further extend this notion to include the chemokine receptor landscape of circulating human monocytes. Using high-dimensional Antibody-based single-cell RNA sequencing (Ab-Seq) of PBMCs, we identified distinct age-associated changes in both C–C and C–X–C chemokine receptor expression profiles in monocyte subsets. Our results identified that both classical and intermediate monocytes exhibited a distinct expression pattern for CCR2, CCR6, CXCR3, CXCR4, and CXCR5 under ‘inflammaging’ conditions. Previous studies have shown that monocytes display distinct expression profiles for chemokine receptors, particularly in healthy individuals (7, 8). Nevertheless, how these monocyte phenotype patterns are altered across age groups and in patients with coronary artery disease is unclear. Our detailed phenotypic profiling revealed age-dependent shifts in monocyte populations associated with high CAD risk, marked by a transition from homeostatic to pro-inflammatory and myeloid-biased immune states in both younger and older individuals.

Conventional flow cytometry approaches have characterized CCR2 expression in aging monocytes (8), yet the roles of other chemokine receptors, for example, CCR5 and CCR6, have been more extensively studied in lymphoid cells (33). However, an association between CCR6 expression and cardiovascular disease in aged monocyte populations has not yet been identified using a high-dimensional Ab-Seq approach. Importantly, in humans, CCR6 has also been widely studied in the context of autoimmune diseases and, to a lesser extent, in the regulation of dendritic cells and B-cells during chronic inflammation (34–36). Recently, studies in animal models have suggested that CCR6 promotes atherosclerosis by regulating monocyte mobilization, adhesion, and recruitment to inflamed vascular sites, leading to increased macrophage accumulation within lesions (34). Those findings are largely based on young or adult mice and do not consider the effects of aging on chemokine receptor-mediated mechanisms. Indeed, previous animal studies have confirmed that chemokine receptor network remodeling was associated with CAD severity (11, 18). In this study, at the subcluster level using computational methods, we identified that cMo_HLA-DR^+^CCR6^+^ and cMo_CD14^low^CXCR3^+^ populations were markedly reduced, whereas iMo_HLA-DR^+^CXCR3^+^ subsets were expanded in older patients with high CAD (**Figure 4G**), indicating selective alterations in chemokine receptor composition in monocytes with age and CAD progression.

The expansion of CXCR3^+^ intermediate monocyte subsets observed in our data is in alignment with our earlier reports showing a marked increase of these subsets in individuals with advanced CAD (9). In this study, we further identify an age-independent expansion of iMo_HLA-DR^+^CXCR3^+^ cells in individuals with high CAD, accompanied by a distinct transcriptomic profile characterized by enrichment of classical complement activation and leukocyte-mediated immunity, together with reduced expression of genes involved in leukocyte adhesion and trafficking (**Figures 4H, I**). The resolution of this transcriptional program across age groups suggests a conserved inflammatory and pro-atherogenic state associated with CAD. Consistent with this observation, prior studies have described C1Q expressing monocytes as a distinct subset marked by elevated complement activity relative to other myeloid cells (37, 38). Also, these C1Q^+^ monocytes have further been linked to altered migratory and adhesive properties and to chronic inflammatory conditions, including atherosclerosis and tissue remodeling (39–41). In this context, our current and previous (9) findings support the idea that C1Q^+^ CXCR3^+^ intermediate monocytes represent an atherogenic monocyte population in CAD. Future studies should define the functional contribution of this subset to vascular inflammation and its role across early and late stages of disease progression.

Integration of monocyte chemokine receptor expression with systemic biochemical parameters revealed close associations with lipid metabolism and inflammatory biomarkers in our data. In older individuals, we identified a positive correlation between CCR6 and CCR5 expression on monocytes with plasma HDL cholesterol, suggesting that chemokine receptor-mediated pathways may influence lipid handling or reverse cholesterol transport (42). On the contrary, our data showed that elevated CXCR5 expression in nonclassical monocytes significantly correlated with LDL cholesterol, implicating its specific role in pro-atherogenic lipid signaling. Interestingly, among the C-X-C family members, only CXCR4 expression (in both iMo and nMo) negatively correlated with hsCRP levels (**Figure 5A**), suggesting a potential inflammatory role for these monocytes. Together, these subset-specific relationships indicate that chemokine receptor remodeling in monocytes integrates lipid metabolism and systemic inflammation, shaping the immune–metabolic interface that may drive cardiovascular disease risk in aging.

Our findings highlight a more complex role of plasma-HDL (mg/dL) in modulating immune function in older adults. While plasma HDL-cholesterol levels are traditionally viewed as atheroprotective, the positive correlation between iMo-specific CCR6 expression and HDL levels in older individuals with high CAD burden (GS>32) challenges this ‘HDL paradigm’ (43–45). Previous studies established that aging is known to alter HDL composition and function, reducing antioxidant capacity and promoting pro-inflammatory signaling through enhanced macrophage lipid uptake (46–48). Our observation (**Figure 5A**) that CCR6 expression on iMo and nMo correlates positively with HDL-cholesterol levels and CAD risk in aged individuals (**Figure 5B**), suggests that dysfunctional HDL may contribute to inflammatory monocyte remodeling rather than protection (48). While our results point to a possible link, definitive evidence confirming that HDL regulates CCR6 expression in human monocyte subsets is still needed. Notably, previous studies have identified a critical role for CCR6 in regulating monocyte trafficking and vascular inflammation in mice. Those findings suggest that CCR6 promotes monocyte–endothelial interactions, leading to enhanced leukocyte accumulation *in vivo* (34). Multiple other studies highlighted the complex and sometimes opposing roles of HDL in cardiovascular and cerebrovascular inflammation. While higher HDL-cholesterol levels have traditionally been associated with a lower incidence of CAD, recent evidence shows that an elevated monocyte-to-HDL cholesterol ratio is linked to microbleeds and the development of cerebral small vessel disease (cSVD) (49–51). Conversely, a recent study by Chang Liu et al. showed that exceptionally high HDL-Cholesterol levels may unexpectedly be linked to an increased risk of mortality among individuals with CAD (52). Taken together, these findings suggest that systemic inflammation, mediated through plasma HDL dynamics, may play a critical role in the pathogenesis of vascular diseases. Here, our findings underscore the need to investigate HDL subtypes and function with monocyte interactions, rather than focusing on HDL quantity alone in evaluating cardiovascular risk in aging populations. This is especially important as we noted that plasma-HDL levels did not differ significantly between older patients either with or without CAD in our cohort. Our findings suggest that future studies should focus on directly assessing the regulatory influence of HDL functionality on monocyte–chemokine signaling.

Our study has several limitations. We did not perform any analyses of atherosclerotic plaque tissue, measure circulating chemokine levels, or conduct functional assays of HDL oxidation or glycation in high CAD risk individuals. We were constrained by limited patient sample availability at the time of this study to further identify sex-based differences across age groups. Also, we did not test whether changes in chemokine receptor expression directly regulate the association between lipid metabolism and CAD severity, which remains an important direction for future mechanistic analysis. Future studies should address these gaps using a larger cohort for comprehensive lipidomic profiling, incorporating functional monocyte assays, and employing spatial imaging-based approaches to characterize plaque features in aging populations. Importantly, for our Ab-Seq analysis, differences between RNA (**Supplementary Figures 4A, B**) and protein expression (**Figures 2B**, **3B**) were expected, as Ab-Seq captures dynamically regulated surface receptor levels, while mRNA expression does not necessarily reflect total or surface protein abundance. These natural biological differences highlight that RNA and protein measurements offer complementary views of cell state. While interpreting RNA and protein differences within the same cell can be challenging, their expression profiles provide valuable insight into regulation beyond transcription and strengthens the biological interpretation of the data rather than raising concerns. Despite these limitations, our findings provide a detailed Ab-Seq–based characterization of chemokine receptor remodeling in circulating monocytes and reveal important associations between immune alterations, lipid metabolism, and the severity of coronary artery disease.

In summary, our findings uncover links between chemokine receptor expression, lipid metabolism, and circulating monocyte profiles, underscoring their potential as biomarkers for cardiovascular risk and therapeutic response. Our observations suggest that dysregulated monocyte-specific chemokine receptor signaling may contribute to both chronic low-grade inflammation and impaired immune surveillance that characterize accelerated inflammaging (53). Therefore, targeted modulation of C–C and C–X–C chemokine receptor pathways in older adults may restore balanced monocyte trafficking, immune homeostasis, and vascular integrity. Together, our findings demonstrate that age, CAD severity reshape the monocyte chemokine receptor axis, providing new insight into immune remodeling and identifying potential therapeutic targets to mitigate cardiovascular disease risk, particularly in older individuals.

## Data Availability

The original contributions presented in the study are included in the article/**Supplementary Material**. Further inquiries can be directed to the corresponding authors.

## Supplementary material

Supplementary Figure 1 t-SNE plots showing all four conditions analyzed in the CAVA cohort across the three major monocyte populations: **(a)** Older High CAD, **(b)** Older Low CAD, **(c)** Younger High CAD, and **(d)** Younger Low CAD. Colors indicate the three major monocyte clusters: classical (cMo), intermediate (iMo), and non-classical (nMo) monocytes.

Supplementary Figure 2 **(A)** t-SNE plots showing all four conditions from the CAVA cohort across all 13 monocyte subclusters, with colors representing individual subclusters. A total of 45,173 cells were included in the t-SNE analyses, comprising 14,304 cells from Older_HighCAD, 10,217 cells from Older_LowCAD, 9,353 cells from Younger_HighCAD, and 11,299 cells from Younger_Low CAD groups. **(B)** Bar plots showing the proportions of all monocyte subclusters in low-risk CAD individuals comparing older and younger age groups; differences are not statistically significant (ns). **(C)** Correlation analysis between age and monocyte subclusters in low CAD risk individuals. **(D)** Volcano plots showing differentially expressed genes across all subclusters in low CAD risk individuals; the y-axis represents −log10(P value) and the x-axis represents log2 fold change (log2FC = 0.1 and min.pct = 0.1).

Supplementary Figure 3 Box plots showing proportions of all monocyte subclusters in younger individuals comparing high and low CAD groups **(A)** and similar analysis for older individuals comparing high and low CAD groups **(B)**; differences are not statistically significant (ns). **C**) Correlation analysis between Gensini score and monocyte subclusters in older individuals, none of them are significant for this Supplementary figure. All significant monocyte subclusters were shown in the main figures.

Supplementary Figure 4 Dot plots showing gene expression profiles of C-C and C-X-C chemokine receptors, along with CD14 (classical monocyte marker) and FCGR3A (non-classical monocyte marker). Scaled average gene expression is shown using a color scale ranging from −1.5 to +3, and the percentage of expressing cells is indicated for each panel. **(A)** Gene expression across the major monocyte populations (cMo, iMo, and nMo). **(B)** Scaled average gene expression across all thirteen subclusters, eight cMo, three iMo and two nMo monocyte subclusters.
