# Supplementary figures and images for: Age-associated chemokine receptor expression profiles in human peripheral blood monocyte subsets predict cardiovascular disease risk
Source: Front Immunol. 2026 Feb 23;17:1749366. doi: 10.3389/fimmu.2026.1749366 (PMC12970611; doi:10.3389/fimmu.2026.1749366)

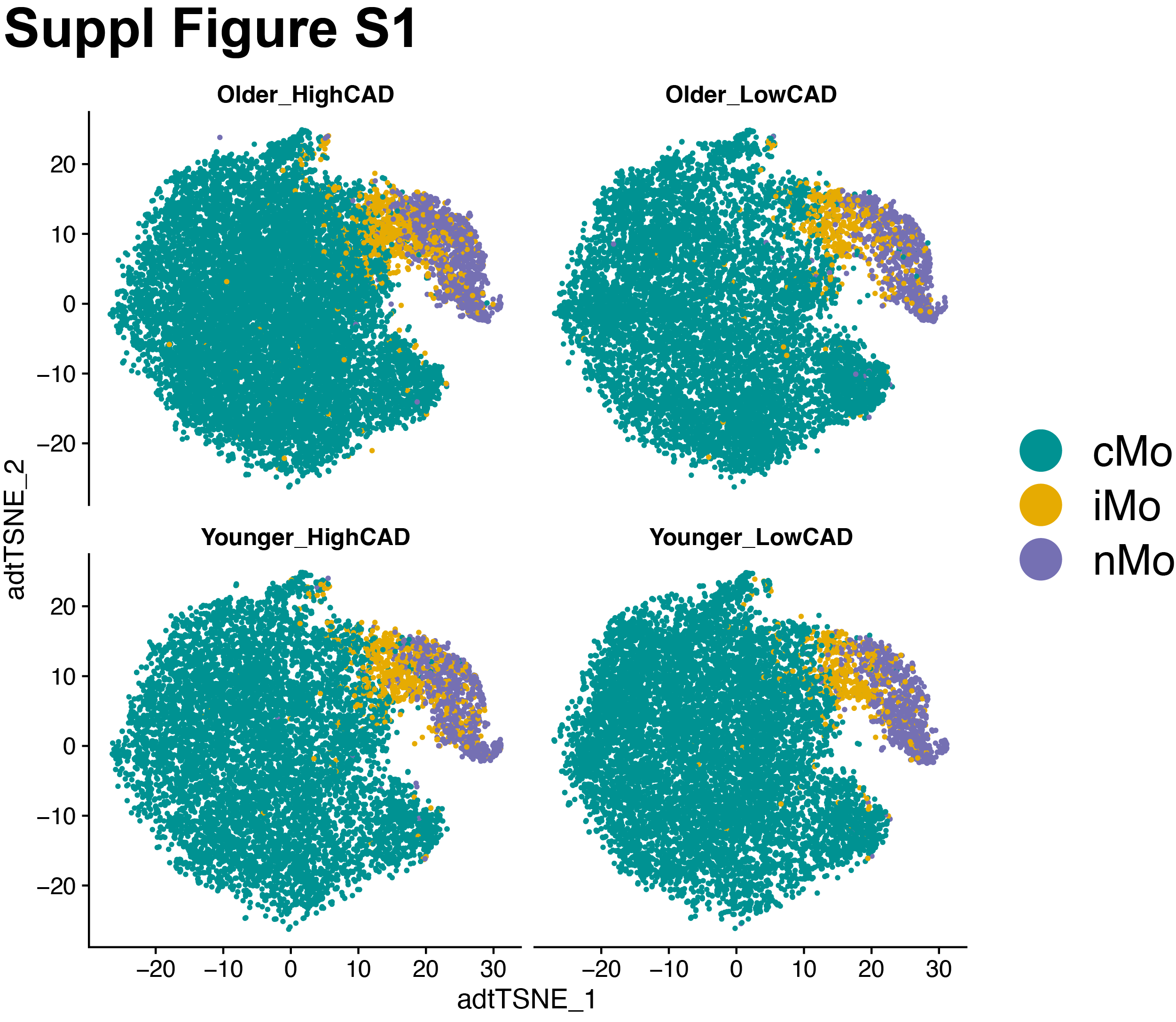

Supplement: Supplementary Figure 1 — t-SNE plots showing all four conditions analyzed in the CAVA cohort across the three major monocyte populations: (a) Older High CAD, (b) Older Low CAD, (c) Younger High CAD, and (d) Younger Low CAD. Colors indicate the three major monocyte clusters: classical (cMo), intermediate (iMo), and non-classical (nMo) monocytes. [file Image1.tif]

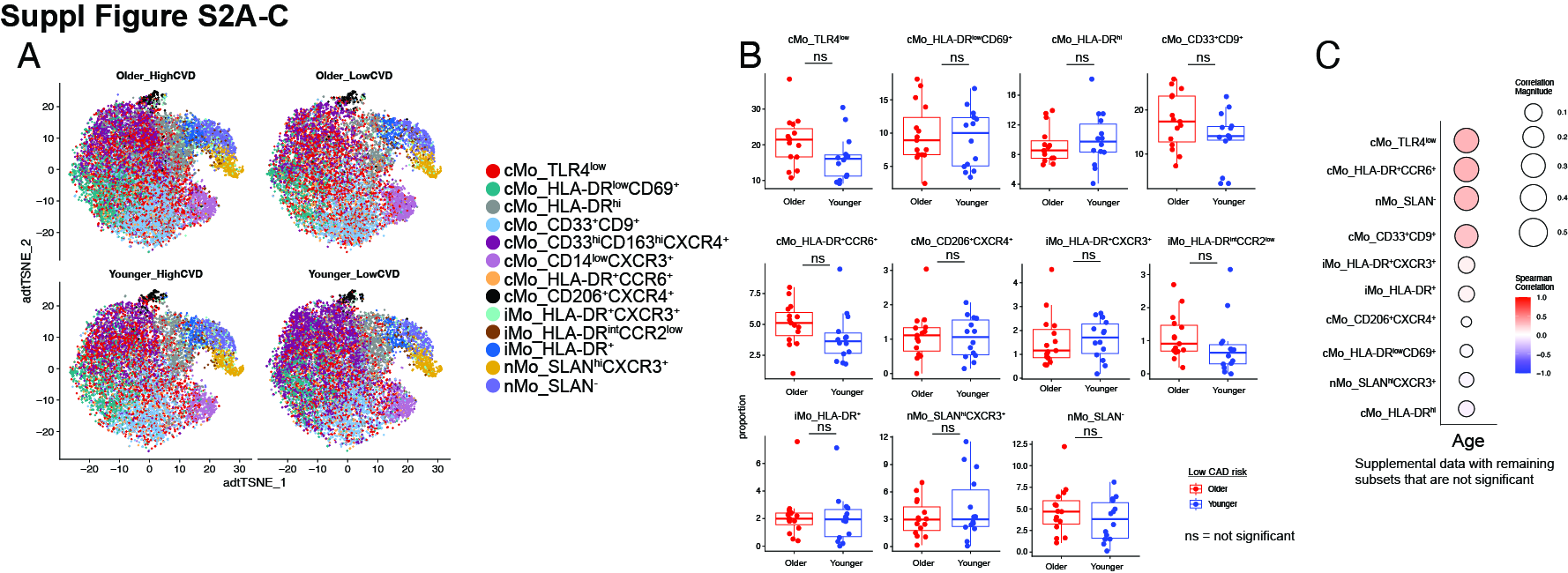

Supplement: Supplementary Figure 2 — (A) t-SNE plots showing all four conditions from the CAVA cohort across all 13 monocyte subclusters, with colors representing individual subclusters. A total of 45,173 cells were included in the t-SNE analyses, comprising 14,304 cells from Older_HighCAD, 10,217 cells from Older_LowCAD, 9,353 cells from Younger_HighCAD, and 11,299 cells from Younger_Low CAD groups. (B) Bar plots showing the proportions of all monocyte subclusters in low-risk CAD individuals comparing older and younger age groups; differences are not statistically significant (ns). (C) Correlation analysis between age and monocyte subclusters in low CAD risk individuals. (D) Volcano plots showing differentially expressed genes across all subclusters in low CAD risk individuals; the y-axis represents −log10(P value) and the x-axis represents log2 fold change (log2FC = 0.1 and min.pct = 0.1). [file Image2.tif]

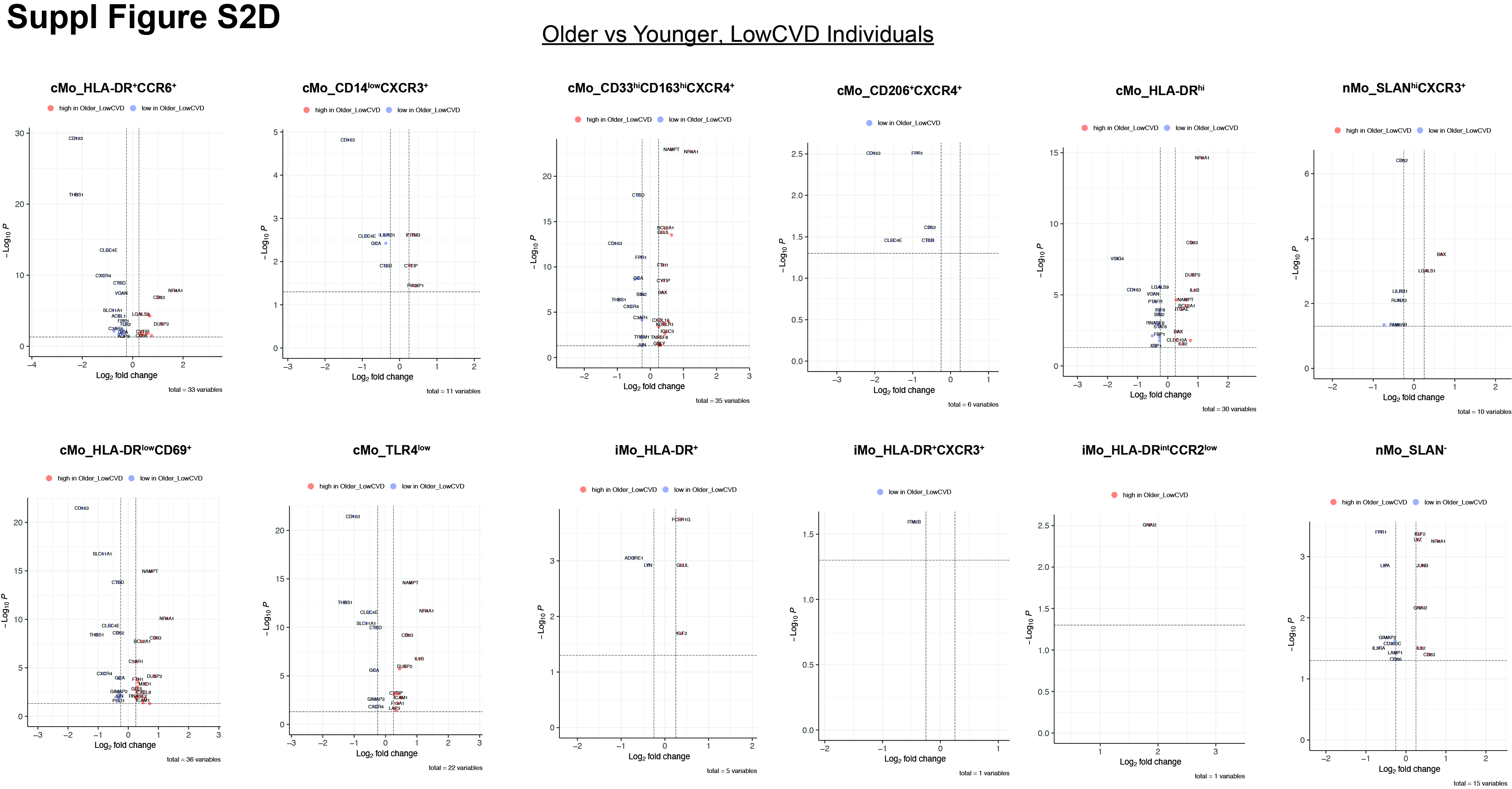

Supplement: Supplementary Figure 3 — Box plots showing proportions of all monocyte subclusters in younger individuals comparing high and low CAD groups (A) and similar analysis for older individuals comparing high and low CAD groups (B); differences are not statistically significant (ns). C) Correlation analysis between Gensini score and monocyte subclusters in older individuals, none of them are significant for this Supplementary figure. All significant monocyte subclusters were shown in the main figures. [file Image3.tif]

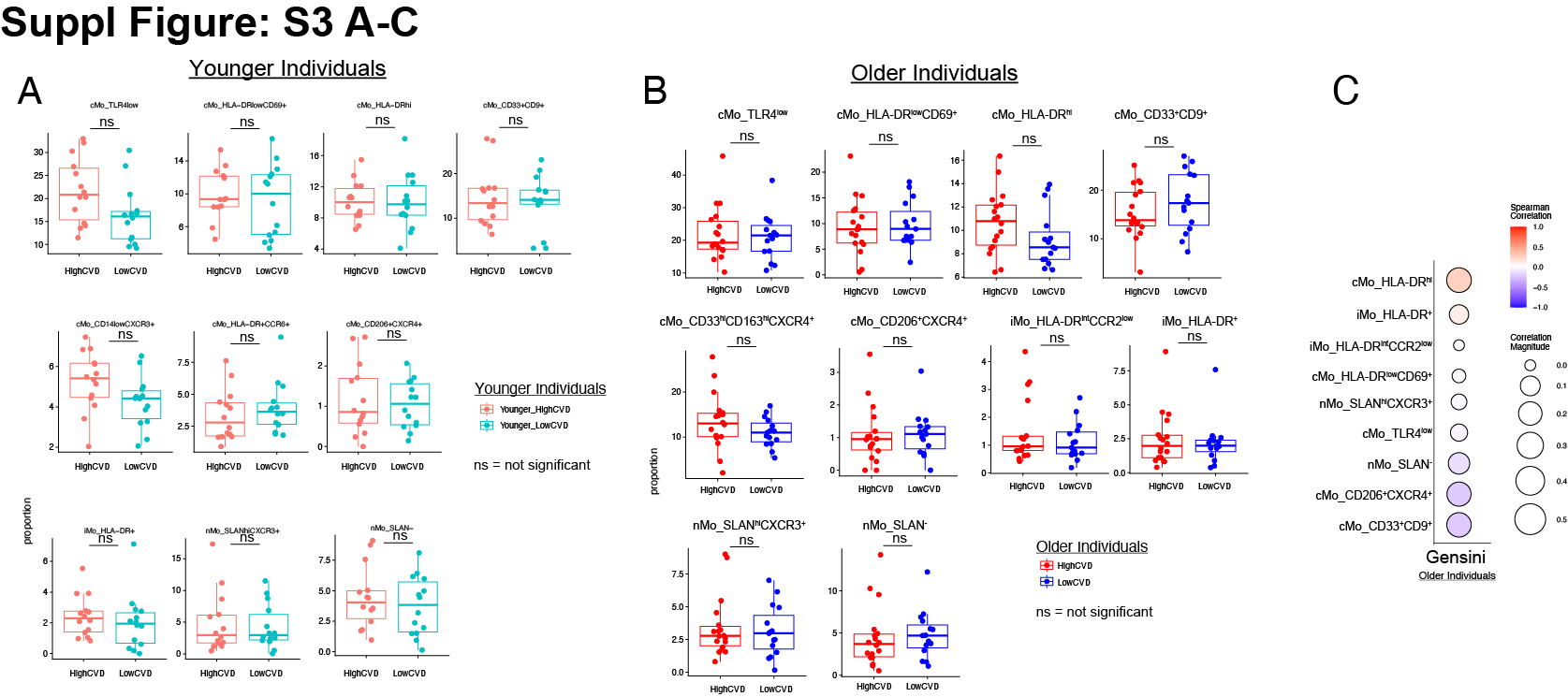

Supplement: Supplementary Figure 4 — Dot plots showing gene expression profiles of C-C and C-X-C chemokine receptors, along with CD14 (classical monocyte marker) and FCGR3A (non-classical monocyte marker). Scaled average gene expression is shown using a color scale ranging from −1.5 to +3, and the percentage of expressing cells is indicated for each panel. (A) Gene expression across the major monocyte populations (cMo, iMo, and nMo). (B) Scaled average gene expression across all thirteen subclusters, eight cMo, three iMo and two nMo monocyte subclusters. [file Image4.tif]

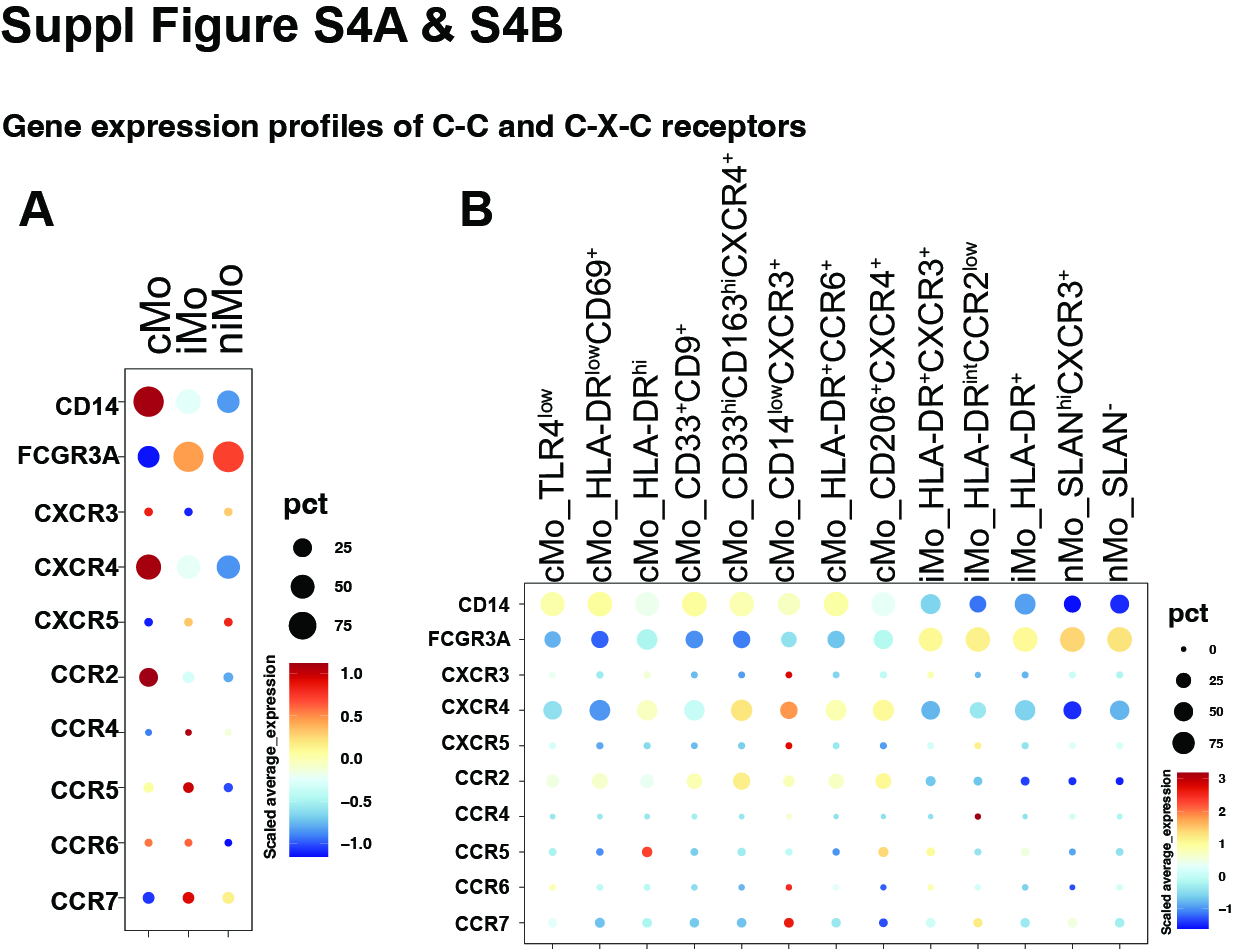

Supplement: Supplementary file 5 [file Image5.tif]
